# Study on Benzylamine(BZA) and Aminoethylpiperazine(AEP) Mixed Absorbent on Ship-Based Carbon Capture

**DOI:** 10.3390/molecules28062661

**Published:** 2023-03-15

**Authors:** Xudong Mao, Hao Chen, Yubing Wang, Xinbo Zhu, Guohua Yang

**Affiliations:** Faculty of Maritime and Transportation, Ningbo University, Ningbo 315832, China

**Keywords:** ship, carbon capture, absorbent, benzylamine, aminoethylpiperazine

## Abstract

To find suitable absorbents for ship-based carbon capture, the absorption and desorption properties of four mixed aqueous amines based on BZA were investigated, and the results indicated that BZA-AEP had the best absorption and desorption performance. Then, the absorption and desorption properties of different mole ratios of BZA-AEP were tested. The results showed that the average CO_2_ absorption rate had the highest value at the mole ratio of BZA to AEP of three. The average CO_2_ desorption rate had the maximum value at the mole ratio of BZA to AEP of one. Three fitted models of the absorption and desorption performance of BZA-AEP based on the test data were obtained. The p-values of all three models were less than 0.0001. Considering the performance and material cost, the BZA-AEP mole ratio of 1.5 is more appropriate for ship carbon capture. Compared with MEA, the average CO_2_ absorption rate increased by 48%, the CO_2_ desorption capacity increased by 120%, and the average CO_2_ desorption rate increased by 161%.

## 1. Introduction

Ship transport is by far the most carbon-efficient mode of commercial transport, and it accounts for about 3% of total global greenhouse gas emissions [1]. An initial strategy was drawn up at the International Maritime Organization (IMO) strategy meeting in April 2018 to peak greenhouse gas emissions from international shipping as soon as possible [2], reducing total annual greenhouse gas emissions by at least 50% by 2050 compared to 2008.

Carbon Capture and Storage (CCS) is one of the most important measures to reduce CO_2_ emissions in international shipping. Luo and Wang [3] estimated the Ship-Based Carbon Capture (SBCC) cost for a cargo ship with a total power of 17 MW. The Post-combustion Carbon Capture (PCC) process integrated with the existing ship energy system can only achieve a 73% carbon capture level. The cost of capturing CO_2_ is about 77.50 EUR/t CO_2_. Feenstra et al. [4] performed ship-based carbon capture simulations for 1280 kW and 3000 kW power ships, and the cost of SBCC for an inland 1280 kW diesel-fueled ship using 30 wt% MEA was 389 EUR/t CO_2_ (60% capture level) and 296 EUR/t CO_2_ (80% capture level). In this case, a capture level of 90% could not be achieved because the exhaust gas from the diesel engine does not provide enough energy for solvent regeneration. Switching to piperazine as a chemical absorption solvent for 1280 kW ships allows the desorption of CO_2_ at higher pressures, thus saving on compression systems and overall costs. For diesel-fueled ships, the costs are 304 EUR/t CO_2_ for a 60% capture level and 207 EUR/t CO_2_ for a 90% capture level. SBCC for 3000 kW LNG-fueled ships costs 120 EUR/t CO_2_ using 30 wt% MEA and 98 EUR/t CO_2_ using piperazine, which can achieve a 90% capture level. However, in the aluminum industry, the cost of carbon capture using MEA is around 55 EUR/t CO_2_ for the flue gas CO_2_ with a concentration of 5% [5]. At the same exhaust gas concentration, the current carbon capture cost in the marine industry is too high compared to other sectors. Therefore, it is necessary to further reduce the cost of SBCC, the core of which is the absorbent selection.

The limited space and residual heat are vital factors limiting SBCC [6]. Therefore, according to voyage type, there are different requirements for the characteristics of marine absorbents. Ships with short voyages may only install absorption towers on the ship and regenerate the rich solvent centrally off the ship. In this case, a high CO_2_ load and fast CO_2_ absorption rate are required for the absorbent. Ships with long voyages need to install a complete carbon capture system (absorption tower, desorption tower, and carbon dioxide storage equipment). Their absorbent requirements are a fast CO_2_ absorption rate, fast CO_2_ desorption rate, high CO_2_ desorption capacity, and low energy consumption.

The absorbent is the key to the carbon capture system. The most widely used method for PCC is chemical absorption. The amine absorbent is the most mature process in the chemical absorption method [7], where ethanolamine (MEA) is the most commonly used. Although MEA has the advantage of a fast CO_2_ absorption rate, it has the disadvantages of easy degradation [8,9], high corrosiveness [10,11,12], and high energy consumption for regeneration. In addition, the International Labor Organization (ILO) said that MEA [13] is harmful to aquatic organisms, and the substance may cause long-term effects on the marine environment.

Amines are classified as primary, secondary, or tertiary according to the number of carbons bonded directly to the nitrogen atom. Primary amines have one carbon bonded to nitrogen. Secondary amines have two carbons bonded to nitrogen, and tertiary amines have three carbons bonded to nitrogen. The reaction process of primary and secondary amines with CO_2_ can be explained by zwitterion [14] and trimolecular [15] mechanisms, while the reaction process of tertiary amines with CO_2_ can be explained by the base-catalyzed hydration mechanism. The reaction (1) of primary amines with CO_2_ and the reaction (2) of secondary amines with CO_2_ mainly produce carbamates. The reaction (3) of tertiary amines with CO_2_ mainly produces bicarbonates, where n represents the number of amino groups in a single molecule. Primary and secondary amines generally react with CO_2_ faster, but their CO_2_ load is lower. Tertiary amines generally react with CO_2_ more slowly, but their CO_2_ load is higher. Therefore, a mixture of both types of amines is considered to combine their advantages.

(1) $${\mathrm{nCO}}_{2}+2{\mathrm{RN}}_{n}H_{2n}\Leftrightarrow {\mathrm{RN}}_{n}H_{n}{\left({\mathrm{COO}}^{-}\right)}_{n}+{\mathrm{RN}}_{n}H_{3n}^{+}$$

(2) $${\mathrm{nCO}}_{2}+2{\mathrm{RN}}_{n}H_{n}\Leftrightarrow {\mathrm{RN}}_{n}{\left({\mathrm{COO}}^{-}\right)}_{n}+{\mathrm{RN}}_{n}H_{2n}^{+}$$

(3) $${\mathrm{nCO}}_{2}+{\mathrm{RN}}_{n}+{\mathrm{nH}}_{2}O\Leftrightarrow R_{1}R_{2}R_{3}N_{n}H^{+}+{\mathrm{nHCO}}_{3}^{-}$$

The published literature indicated that Benzylamine (BZA) has better absorption performance than MEA. Richner et al. [16] evaluated the facilitation of MDEA by MEA, DEA, and BZA. The results showed that the CO_2_ absorption rate of MDEA increased by BZA was the highest. Conway et al. [17] measured the mass transfer coefficients of MEA and BZA. They found that the mass transfer coefficient of BZA was more excellent at the same concentration and the difference between BZA and MEA was more significant as the concentration increased. Richner et al. [18] observed that BZA has similar reaction kinetics to MEA. BZA has a larger negative enthalpy, which is usually favorable for the absorption rate [19]. However, BZA absorbs CO_2_ to form precipitates at high concentrations (≥50 wt%). Gao et al. [20] identified that the mixture of MEA and BZA would form a precipitate with a high CO_2_ load. Mukherjee et al. [21] used an artificial neural network (ANN) model to predict the CO_2_ solubility of a mixture of BZA and N-(2-aminoethyl)-ethanolamine (AEEA). Zheng et al. [22] investigated the reaction kinetics of BZA with CO_2_ using a stopped-flow apparatus. They showed that BZA has a higher secondary reaction rate and lower activation energy compared to MEA, diethanolamine (DEA), methyl diethanolamine (MDEA), and 2-amino-2-methyl-1-propanol (AMP). The values predicted by the zwitterion and the termolecular mechanism models were compared with the experimental values with absolute average deviation (AAD) of 5.15% and 4.15%, respectively. Both models could be used to explain the reaction kinetics of BZA with CO_2_. Puxty et al. [23] studied the effect of nine co-solvents on the vapor pressure of BZA, among which imidazole was the most effective in reducing the vapor pressure of BZA. Chen et al. [24] examined the performance of BZA mixed with MEA. The CO_2_ load of BZA-MEA mixed absorbent with different concentration ratios did not vary significantly, and BZA should not exceed 3M in the starting solution to avoid the formation of white precipitation, which would hinder further absorption of CO_2_.

Theoretically, BZA has lower energy consumption than MEA for desorption. The heat capacity of BZA [25] (25 °C, 1.93 J·g^−1^·K^−1^) is smaller than MEA [26] (30 °C, 2.74 J·g^−1^·K^−1^). Through theoretical calculations, Mukherjee et al. [27,28,29] concluded that BZA has a small reaction energy potential barrier (∼26 kJ/mol). Later, the heat of absorption and heat capacity of BZA, AEEA, and their mixtures in aqueous solutions were measured using an automated reaction calorimeter, and the results showed that the solution heat capacity increased with increasing temperature. Moreover, for the BZA-AEEA mixture, the solution heat capacity increased with the larger percentage of AEEA concentration.

The aromatic structure makes BZA low-corrosive and highly stable. Martin et al. [30] compared the corrosiveness and stability of 22 amines. They noted that the MEA solution was highly corrosive to carbon steel and stainless steel, while BZA was less corrosive. The degradation rate of MEA was more than twice that of BZA after 14 days at 140 °C and 0.5 MPa with the influx of a gas mixture of 75% CO_2_, 20% N_2,_ and 5% O_2_. Because aromatic compounds form a protective film on metal surfaces, they are considered intrinsically non-corrosive [31]. BZA is excellently biodegradable [32], with 96.1–98.9% degradation after six days in lake water [33].

Although the available literature indicates that BZA has the advantages of a fast CO_2_ absorption rate, low heat capacity, high stability, low corrosiveness, and easy biodegradation, BZA has a lower desorption capacity like MEA. Ship-based carbon capture absorbents need to have a large desorption capacity. Therefore, this paper examines the absorption and desorption properties of four mixed aqueous amines based on BZA to further increase the desorption capacity of BZA.

## 2. Results and Discussion

### 2.1. Absorption and Desorption Properties of Mixed Aqueous Amines Based on BZA

BZA, as the primary amine, has a fast CO_2_ absorption rate, but its CO_2_ desorption capacity is relatively low. Tertiary amines and steric hindrance amines have low CO_2_ absorption rates and high CO_2_ desorption capacity. For this reason, four amines (DMEA, DEEA, AEP, and AMP) were selected to improve the CO_2_ desorption capacity of BZA. The absorption and desorption performance of the four mixed aqueous amines based on BZA was investigated with a total amine concentration of 3 mol/kg and a ratio of 2:1 between BZA and each of the four amines.

**Figure 1:**
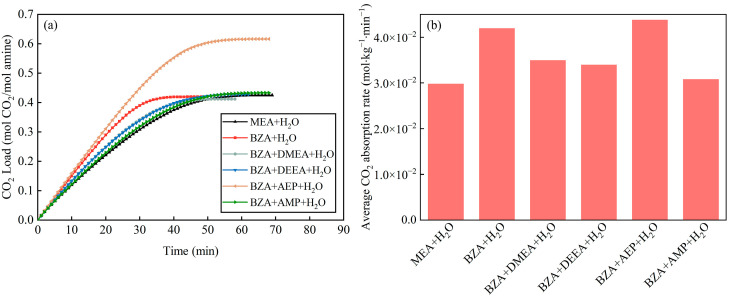
Absorption characteristics of mixed aqueous amines (**a**) the change in the CO_2_ load of the mixed aqueous amines and (**b**) the average CO_2_ absorption rate of the mixed aqueous amines.

According to the change in the CO_2_ load of the mixed aqueous amines, it can be seen in Figure 1a that the mixture of BZA-AEP increased the CO_2_ load by 45% relative to MEA and 47% relative to BZA. Compared to BZA, the other three mixed aqueous amines did not increase the CO_2_ load much because DMEA, DEEA, and AMP all have only one amino group per molecule for CO_2_ fixation, while AEP has three amino groups per molecule. As can be seen in Figure 1b, the BZA absorption rate is faster than MEA, and BZA has a larger negative CO_2_ absorption enthalpy than typical amines due to its structural rigidity [31], which facilitates the absorption reaction. When the lone pair of electrons of the nitrogen on the amino group is distributed to form a bond, the reduction of the supplied electron sites will limit the activity of the reaction of the amine with carbon dioxide [34]. In contrast, the lone pair of electrons on the ammonia atom of BZA is not delocalized into the Π system of the benzene ring by the presence of the carbon atom [18]. The average CO_2_ absorption rates of BZA-DMEA, BZA-DEEA, BZA-AEP, and BZA-AMP were increased by 17%, 14%, 47%, and 3%, respectively, relative to MEA. Relative to BZA, the average CO_2_ absorption rates of BZA-DMEA, BZA-DEEA, and BZA-AMP decreased to different extents. Among them, the average CO_2_ absorption rate of the BZA-AMP absorbent decreased the most. The reason for this is the formation of intramolecular hydrogen bonds in solution, when the lone pair of electrons of nitrogen is distributed into bonds, the supply electron sites are reduced, which limits the activity of amine reaction in CO_2_ [34]. AMP makes the carbamate unstable due to the steric hindrance effect, which limits the CO_2_ absorption rate. The average CO_2_ absorption rate of BZA-AEP absorbent was slightly increased compared with that of BZA because AEP has three amino groups, increasing the reaction site with CO_2_ [35]. No precipitation was produced during the absorption experiments.

**Figure 2:**
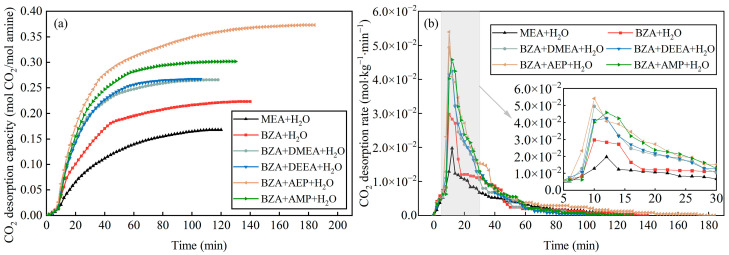
Desorption characteristics of mixed aqueous amines. (**a**) the variation of mixed aqueous amine CO_2_ desorption capacity and (**b**) the variation of mixed aqueous amine average CO_2_ desorption rate.

**Figure 3:**
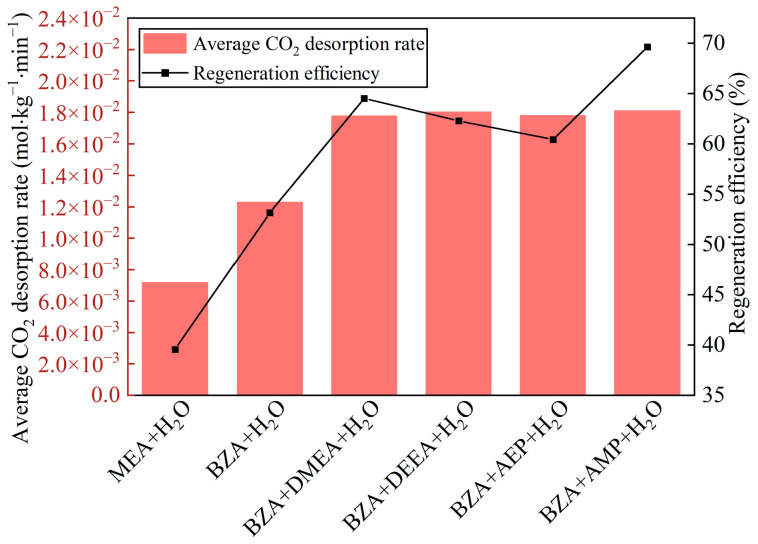
Average desorption rate and regeneration efficiency of mixed aqueous amine.

According to the variation of mixed aqueous amine CO_2_ desorption capacity, it can be seen from Figure 2a that the equilibrium desorption time was shortened for BZA-DMEA, BZA-DEEA, and BZA-AMP and extended for BZA-AEP relative to BZA. BZA-AEP has the largest CO_2_ desorption capacity, reaching 0.373 mol CO_2_/mol amine, which improved by 122% compared to MEA and 67% to BZA. The CO_2_ desorption capacity of BZA-DMEA, BZA-DEEA, and BZA-AMP was enhanced by 58%, 59%, and 79%, respectively, in contrast to MEA and by 19%, 20%, and 35%, respectively, in comparison to BZA. The carbamate formed by tertiary amines after absorbing carbon dioxide is less stable than primary amines. At the same time, according to Sartori and Savage [36], the compound is susceptible to hydrolysis reactions of carbamates due to steric hindrance effects. A large number of substitutions in the steric hindrance amine will reduce the stability of the carbamate and thus achieve a higher CO_2_ desorption capacity [37]. As shown in Figure 2b, the CO_2_ desorption rate of the absorbents gradually increased in the first 10 min because the desorption experiment started from 50 °C with a certain heating time to reach the desorption temperature. Then, the CO_2_ desorption rate began to decrease as the CO_2_ load in the solution gradually reduced. The maximum CO_2_ desorption rate of BZA-AEP increased the most, by 173% over MEA and 81% over BZA. In comparison to MEA, BZA-DMEA, BZA-DEEA, and BZA-AMP, the maximum CO_2_ desorption rates grew 150%, 114%, and 132%, respectively. Compared to BZA, the maximum CO_2_ desorption rates increased by 66%, 42%, and 54%, respectively. The CO_2_ desorption rates did not change much until 10 min and changed very minimally after 60 min. In Figure 3, it is apparent that adding DMEA, DEEA, AEP, and AMP increases the average CO_2_ desorption rate of BZA. In addition, the degree of improvement is basically the same, which is about 150% compared to MEA and about 45% compared to BZA. Moreover, it was found that four mixed aqueous amines had a regeneration efficiency of around 65%. The reason for the high CO_2_ desorption capacity and high CO_2_ desorption rate of BZA-AEP may be the presence of steric hindrance in branched alkanolamines, which results in faster desorption rates, higher cycle capacities, and lower regeneration heat loads than the straight-chain amine analogs [38].

### 2.2. Effect of BZA and AEP Concentration Ratio

A comparison of the absorption and desorption performance of four mixed aqueous amines based on BZA revealed that BZA-AEP performed better. The experiments were designed using the simplex lattice design method to investigate the effect of different concentration ratios of BZA-AEP on both absorption and desorption performance. A description of the experimental design can be found in Table 1.

#### 2.2.1. Absorption and Desorption Performance

**Figure 4:**
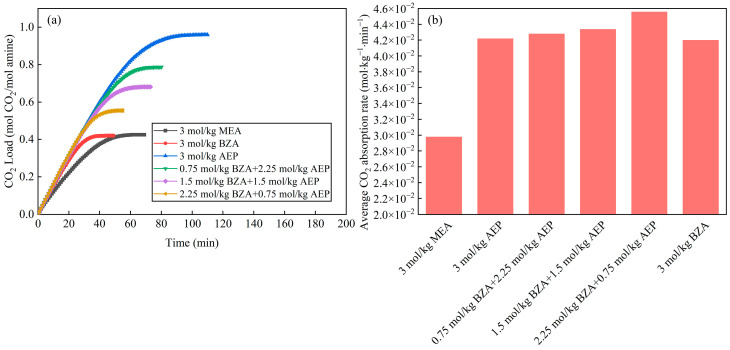
Absorption characteristics of BZA-AEP. (**a**) the effects of the BZA-AEP concentration ratio on CO_2_ load and (**b**) the effects of the BZA-AEP concentration ratio on average CO_2_ absorption rate.

The effects of the BZA-AEP concentration ratio on CO_2_ load are shown in Figure 4a, which shows that a higher BZA concentration results in a shorter absorption equilibrium time, while a higher AEP concentration results in a higher CO_2_ load. The CO_2_ load and absorption equilibration time have excellent linear relationships with the concentration ratio. The CO_2_ load of BZA-AEP increased by 30~85% relative to MEA. Figure 4b shows that the average CO_2_ absorption rate of BZA-AEP shot up by 41~53% relative to MEA. With an increase in BZA concentration, the average CO_2_ absorption rate increased and then decreased. A BZA to AEP concentration ratio of about three was the most optimal. No precipitation was found in the absorption process when observing different concentration ratios of BZA-AEP absorbents. Zhang et al. [39] investigated the CO_2_ absorption and desorption performance of a mixed aqueous amine of MEA, N-methyldiethanolamine (MDEA), and piperazine (PZ) as a CO_2_ capture solvent. The total concentration of the mixed aqueous amine was 6M, mixing different amine molar ratios. Among them, 3M MEA-1.5M MDEA-1.5M PZ showed the best absorption and desorption performance, the average CO_2_ absorption rate was increased by 20% compared with 5M MEA. The result is obvious; compared with 3M MEA-1.5M MDEA-1.5M PZ, the average CO_2_ absorption rate of BZA-AEP is more improved than conventional absorbent (MEA).

**Figure 5:**
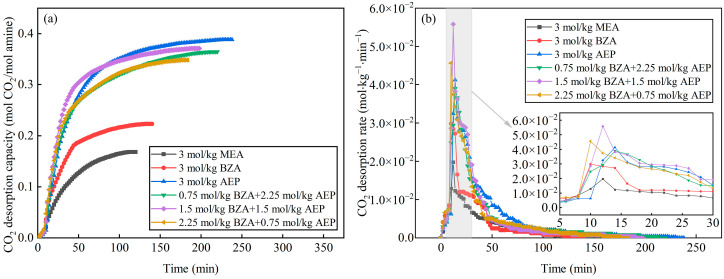
Desorption characteristics of BZA-AEP. (**a**) the effects of the BZA-AEP concentration ratio on CO_2_ desorption capacity and (**b**) the effects of the BZA-AEP concentration ratio on CO_2_ desorption rate.

**Figure 6:**
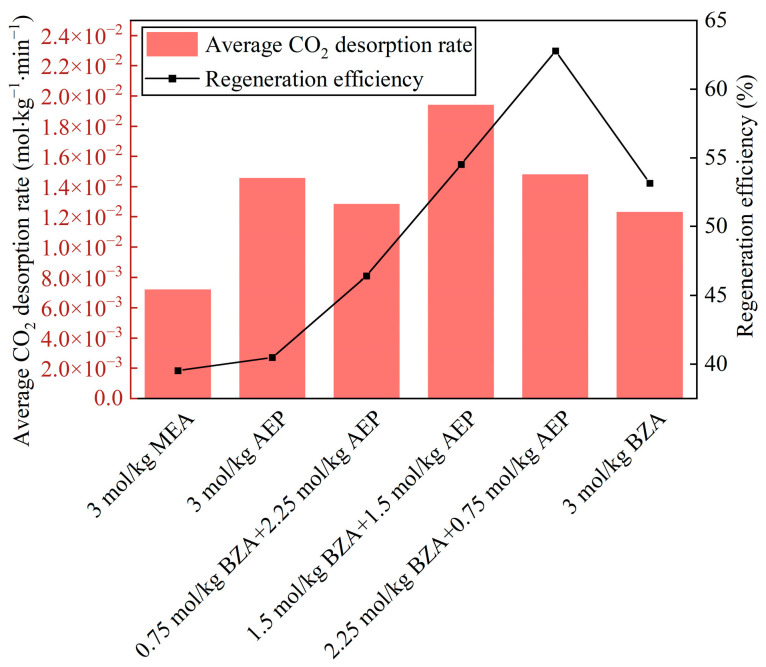
Average desorption rate and regeneration efficiency of BZA-AEP.

The BZA-AEP concentration ratio affects CO_2_ desorption capacity and rate. Figure 5a illustrates the variation in the CO_2_ desorption capacity of BZA-AEP with time. The desorption equilibrium time increases as the proportion of AEP concentration increases. Although AEP has a high CO_2_ load, most of it cannot be desorbed, and mixing it with BZA enhances its regeneration efficiency. The CO_2_ desorption capacity of BZA-AEP is increased by 33~131% relative to MEA. Moreover, the thermal degradation of AEP is relatively high. In Figure 5b, it is shown that the time to reach the maximum CO_2_ desorption rate decreases with increasing BZA concentration. The maximum CO_2_ desorption rate of BZA-AEP increased by 51% to 182% relative to MEA. It can be seen from Figure 6 that the average CO_2_ desorption rate of BZA-AEP was increased by 70~170% compared to MEA, which had the highest value at a BZA to AEP concentration ratio of about one. The average CO_2_ desorption rate of 3M MEA-1.5M MDEA-1.5M PZ mixed aqueous amines studied by Zhang et al. [39] was 119% faster than that of 5M MEA. When the concentration ratio of BZA to AEP was one, the average CO_2_ desorption rate of BZA-AEP was more enhanced than that of traditional absorbent MEA relative to 3M MEA-1.5M MDEA-1.5M PZ. The highest regeneration efficiency is 63%, which has the largest value at a BZA to AEP concentration ratio of about three.

#### 2.2.2. Fitted Model

Among the five indicators of absorption and desorption performance, the average CO_2_ absorption rate, average CO_2_ desorption rate, and CO_2_ desorption capacity are the most important. According to the test points designed in Table 1, the three response indicators of average CO_2_ absorption rate (Y_1_), average CO_2_ desorption rate (Y_2_), and CO_2_ desorption capacity (Y_3_) were fitted with polynomials to explore the relationship between BZA concentration (X_1_) and AEP (X_2_) concentration and these three.

In the model for the average CO_2_ absorption rate, its F-value of 513.19 implies the model is significant (*p*-value < 0.0001). In this case $X_{1}$, $X_{2}$, $X_{1}X_{2}$, $X_{1}^{2}X_{2}$, $X_{1}X_{2}^{2}$, $X_{1}^{3}X_{2}$, $X_{1}^{2}X_{2}^{2}$, and $X_{1}X_{2}^{3}$ are significant model terms. The ‘Adj R-Squared’ of 0.9966 is in reasonable agreement. The model for the average CO_2_ desorption rate has an F-value of 1421.48, which indicates that the model is significant (*p*-value < 0.0001). There are significant model terms associated with $X_{1}$, $X_{2}$, $X_{1}X_{2}$, $X_{1}^{2}X_{2}$, $X_{1}X_{2}^{2}$, $X_{1}^{3}X_{2}$, $X_{1}^{2}X_{2}^{2}$, and $X_{1}X_{2}^{3}$ in this case. The ‘Adj R-Squared’ of 0.9988 is in reasonable agreement. Considering the F-value of 2277.16 for the model of CO_2_ desorption capacity, it is evident that the model is significant (*p*-value < 0.0001). It should be noted that model terms $X_{1}$, $X_{2}$, $X_{1}X_{2}$, $X_{1}^{2}X_{2}$, and $X_{1}X_{2}^{2}$ are significant in this case. The ‘Adj R-Squared’ of 0.9990 is in reasonable agreement.

**Figure 7:**
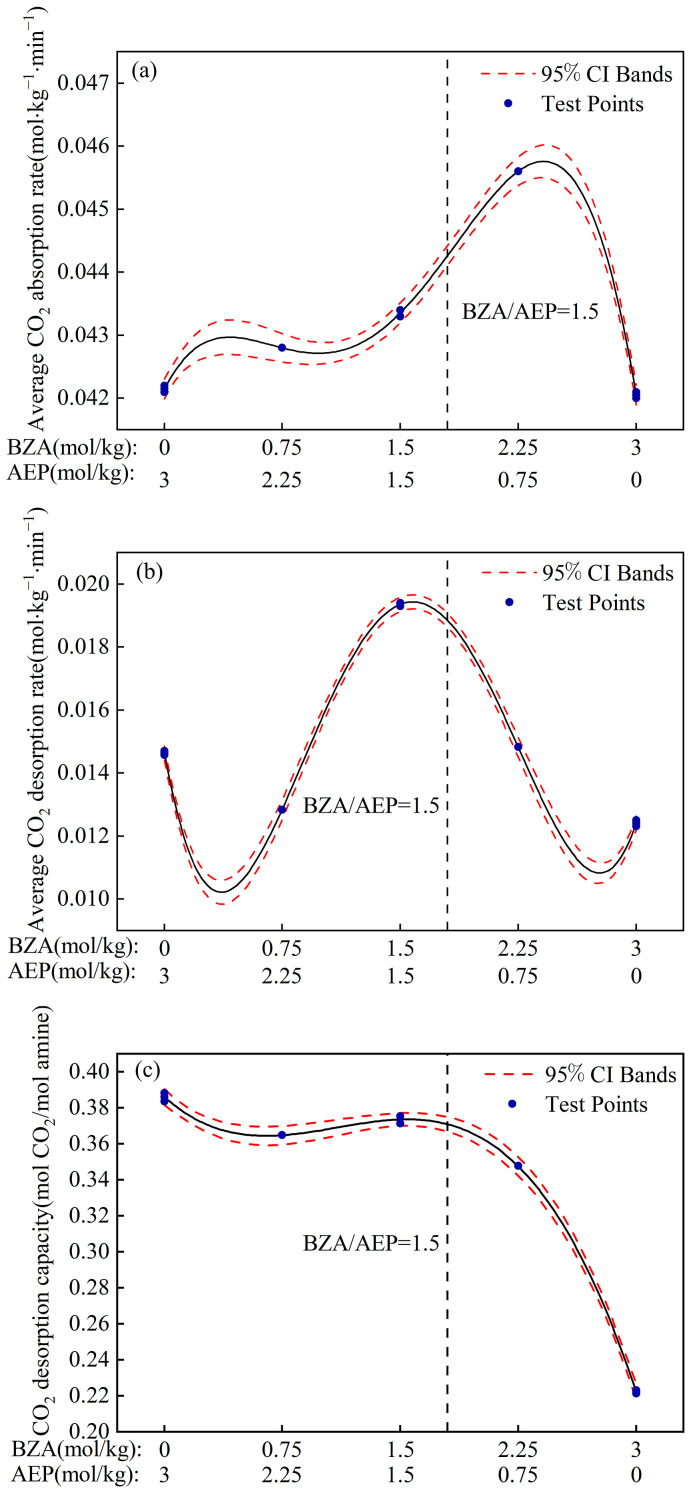
Fitted model of BZA-AEP absorption and desorption performance. (**a**) the average CO_2_ absorption rate, (**b**) the average CO_2_ desorption rate and (**c**) the CO_2_ desorption capacity.

Figure 7 shows the fitted model for the three response indicators. It can be seen that the absorption and desorption performance is non-linearly related to the concentration ratio of BZA-AEP. There is an interaction between BZA and AEP. The average CO_2_ absorption rate has a maximum value when BZA/AEP is around three. The average CO_2_ desorption rate has a maximum value when BZA/AEP is about one. The price of AEP is 68% higher than that of BZA, and the higher the AEP concentration, the higher the absorbent costs. If a ship needs to install a complete carbon capture system, it is more economical to have a BZA/AEP of about 1.5, and its regeneration efficiency is about 55%. Compared with MEA, the average CO_2_ absorption rate increases by 48%, the CO_2_ desorption capacity increases by 120%, and the average CO_2_ desorption rate increases by 161%.

## 3. Materials and Methods

### 3.1. Materials

**Figure 8:**
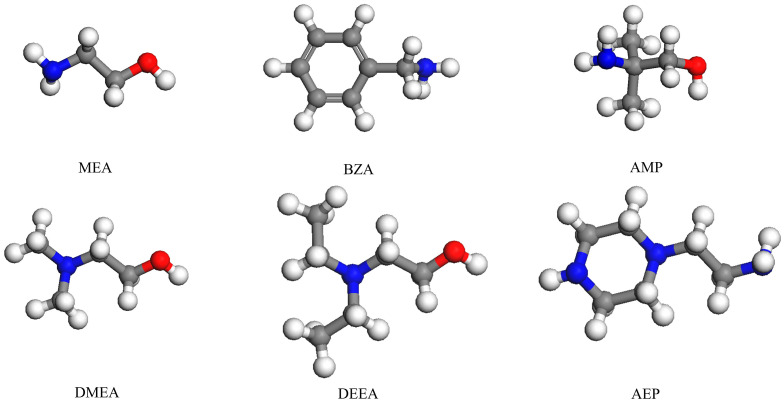
Structure of amines in this study.

Ethanolamine (MEA, 141-43-5, Xiya, 99%), benzylamine (BZA, 100-46-9, Macklin, 99%), 2-amino-2-methyl-1-propanol (AMP, 124-68-5, Macklin, 99%), N, N-dimethylethanolamine (DMEA, 108-01-0, Macklin, 99%), N, N-diethylethanolamine (DEEA, 100-37-8, Macklin, 99%), aminoethylpiperazine (AEP, 140-31-8, Macklin, 99%), deionized water (DI, Macklin), CO_2_ gas (Ningbo Fangxin Gas Company, Ningbo, China, 99.9%), and N_2_ gas (Ningbo Fangxin Gas Company, Ningbo, China, 99.9%) were purchased directly and without further purification. Table 2 shows the physicochemical properties of these reagents. Figure 8 shows the structure of amines.

### 3.2. Experimental Setup

**Figure 9:**
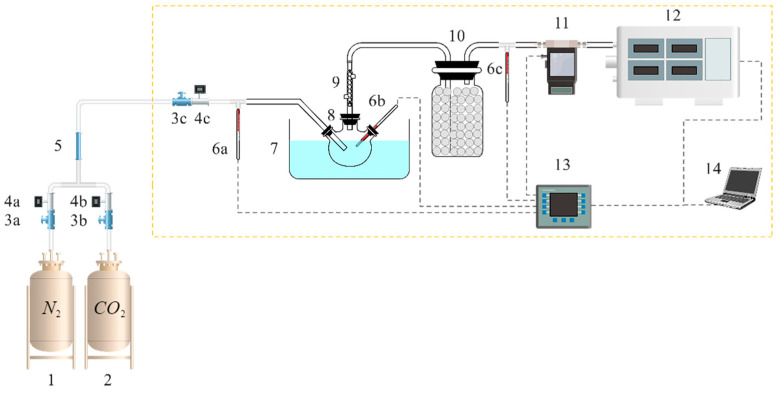
Flow chart of the experimental setup (1. N_2_ cylinder, 2. CO_2_ cylinder, 3. flow control valve (3a,3b,3c), 4. gas flow meter (4a,4b,4c), 5. gas mixer, 6. temperature sensor (6a,6b,6c), 7. oil bath, 8. three-neck flask, 9. condenser tube, 10. drying bottle, 11. mass flow meter, 12. flue gas analyzer, 13. data acquisition instrument, 14. computer).

The experimental setup is shown in Figure 9. A flue gas analyzer (testo 350, Testo SE & Co. KGaA, Titisee-Neustadt, Germany) was used to detect the outlet CO_2_ concentration with a sampling flow of 1 L/min; a data acquisition instrument (DAQ970A, Keysight, Santa Rosa, CA, America) was used to collect the inlet and outlet gas temperatures, the solution temperature, and the outlet gas flow. Before the experiment, a leak test and N_2_ purge were performed on the system. According to the ship exhaust treatment process, the flue gas temperature is around 50 °C, and the CO_2_ concentration is about 5% after denitrification, dust removal, and desulfurization [1,40]. For the absorption experiments, the valves 3a, 3b, and 3c were opened, the inlet CO_2_ concentration was 5%, the inlet gas flow was 1.25 L/min, the absorption temperature was 50 °C, and the absorption solution was 50 g. The valves 3a, 3b, and 3c were closed for the desorption experiments, and the desorption temperature was 100 °C. Each group of experiments was repeated three times, and the results were averaged three times.

### 3.3. Data Processing

There are five primary absorption and desorption performance indicators of absorbents, which are CO_2_ load, CO_2_ absorption rate, CO_2_ desorption capacity, CO_2_ desorption rate, and regeneration efficiency. Currently, the CO_2_ load of the rich solvent in the engineering carbon capture system is about 80% of its equilibrium load [41,42,43,44]. Therefore, the average absorption rate in this paper is the average CO_2_ absorption rate when the absorbent reaches 80% of the equilibrium load, and the average desorption rate is the average CO_2_ desorption rate when the absorbent comes to 80% of the equilibrium desorption capacity. The following are the formulas for calculating these indicators.

(4) $$m_{i}=273.15\times \frac{44}{22.4}\times \left(\frac{V_{0}c_{0}t}{T_{0}}-\sum \frac{V_{i}c_{i}∆t}{T_{i}}\right)$$

(5) $$\alpha_{i}=\frac{m_{i}/44}{\sum m_{j}/M_{j}}$$

(6) $$v_{i}^{\mathrm{abs}}=\frac{dm_{i}}{44m_{s}∆t}$$

where $m_{i}$ is CO_2_ absorption capacity at the moment *i* (g), $\alpha_{i}$ is CO_2_ load at the moment *i* (mol CO_2_/mol amine), $m_{j}$ is the mass of component *j* of the amine in the absorbent to be measured (g), $M_{j}$ is the molar mass of component *j* of the amine in the absorbent to be measured (g/mol), $V_{0}$ is the gas inlet flow (L/min), $c_{0}$ is inlet CO_2_ concentration (%), $V_{i}$ is the gas outlet flow at the moment *i* (L/min), $c_{i}$ is outlet CO_2_ concentration at the moment *i* (%), *t* is reaction time (min), ∆t is recording time step (min), $T_{0}$ is gas inlet temperature (K), $T_{i}$ is gas outlet temperature at the moment *i* (K), $v_{i}^{\mathrm{abs}}$ is CO_2_ absorption rate at the moment *i* (mol·kg^−1^·min^−1^), and $m_{s}$ is the mass of the solution (kg).

(7) $$\beta_{i}=\frac{\sum 273.15V_{i}c_{i}∆t/T_{i}}{22.4\sum m_{j}/M_{j}}$$

(8) $$v_{i}^{des}=\frac{273.15V_{i}c_{i}}{22.4m_{s}T_{i}}$$

(9) $$\eta_{i}=\frac{\beta_{i}}{\alpha_{bal}}\times 100\%$$

where $\beta_{i}$ is CO_2_ desorption capacity from 0 to *i* (mol CO_2_/mol amine), $v_{i}^{des}$ is CO_2_ desorption rate at the moment *i* (mol·kg^−1^·min^−1^), $\eta_{i}$ is regeneration efficiency at the moment *i* (%), and $\alpha_{bal}$ is the CO_2_ equilibrium load (mol CO_2_/mol amine).

## 4. Conclusions

1. The absorption and desorption performance of four mixed aqueous amines based on BZA was investigated, and it was found that BZA-AEP had the highest average CO_2_ absorption rate and CO_2_ desorption capacity. The CO_2_ absorption rate of BZA-DMEA, BZA-DEEA, and BZA-AMP decreased in comparison to BZA under the same conditions, while the CO_2_ absorption rate of BZA-AEP did not decrease. In contrast to MEA, the average CO_2_ absorption rate of BZA-AEP increased by 47%, and the CO_2_ desorption capacity increased by 122%. By adding DMEA, DEEA, AEP, and AMP to BZA, the average CO_2_ desorption rate was enhanced by about 150% compared with MEA and by about 45% compared with BZA.
2. The absorption and desorption characteristics of BZA-AEP with different concentration ratios were investigated. The results indicated that there was an optimal concentration ratio for the average CO_2_ absorption rate and average CO_2_ desorption rate. The average CO_2_ absorption rate of BZA-AEP improved by 41~53%, the CO_2_ desorption capacity improved by 33~131%, and the average CO_2_ desorption rate improved by 70~170% relative to MEA. It was found that the CO_2_ load and the absorption equilibrium time had a linear relationship with the concentration ratio, and the desorption equilibrium time increased with the proportion of AEP in the solution.
3. The model established by the experimental data indicates that it is more economical to install a complete set of carbon capture systems on a ship with a BZA/AEP concentration ratio of approximately 1.5. Compared with MEA, its average CO_2_ absorption rate increases by 48%, its CO_2_ desorption capacity increases by 120%, and its average CO_2_ desorption rate increases by 161%. In light of its excellent absorption and desorption characteristics, BZA-AEP mixed aqueous amine can reduce the design size of the absorption tower and desorption tower to solve the problem of limited space for SBCC. Therefore, BZA-AEP can be considered a candidate material for SBCC.
4. The regeneration efficiency of BZA-AEP is around 55%, and there is still much space to improve its desorption performance using a solid acid catalyst in the future.

## Figures and Tables

**Table 1:** Experimental design.

Std	Run	BZA (mol/kg)	AEP (mol/kg)
6	1	3	0
2	2	0	3
1	3	3	0
3	4	1.5	1.5
5	5	0.75	2.25
7	6	0	3
8	7	1.5	1.5
4	8	2.25	0.75

**Table 2:** Physicochemical properties of materials.

Reagent Name	Abbreviation	Boiling Point/°C (101.325 kPa)	Dynamic Viscosity/mPa·s (20 °C)
ethanolamine	MEA	170	7.5
benzylamine	BZA	185	1.78
2-amino-2-methyl-1-propanol	AMP	165	147
N, N-dimethylethanolamine	DMEA	134	3.58
N, N-diethylethanolamine	DEEA	163	5
aminoethylpiperazine	AEP	220	15.4

## Data Availability

Not applicable.
